# A Novel Tool for the Identification and Characterization of Repetitive Patterns in High-Density Contact Mapping of Atrial Fibrillation

**DOI:** 10.3389/fphys.2020.570118

**Published:** 2020-10-15

**Authors:** Stef Zeemering, Arne van Hunnik, Frank van Rosmalen, Pietro Bonizzi, Billy Scaf, Tammo Delhaas, Sander Verheule, Ulrich Schotten

**Affiliations:** ^1^Department of Physiology, Maastricht University Medical Center, Cardiovascular Research Institute Maastricht, Maastricht, Netherlands; ^2^Department of Biomedical Engineering, Maastricht University Medical Center, Cardiovascular Research Institute Maastricht, Maastricht, Netherlands; ^3^Department of Data Science and Knowledge Engineering, Maastricht University, Maastricht, Netherlands

**Keywords:** atrial fibrillation, mapping, recurrence plots, repetitive conduction patterns, mechanisms

## Abstract

**Introduction:**

Electrical contact mapping provides a detailed view of conduction patterns in the atria during atrial fibrillation (AF). Identification of repetitive wave front propagation mechanisms potentially initiating or sustaining AF might provide more insights into temporal and spatial distribution of candidate AF mechanism and identify targets for catheter ablation. We developed a novel tool based on recurrence plots to automatically identify and characterize repetitive conduction patterns in high-density contact mapping of AF.

**Materials and Methods:**

Recurrence plots were constructed by first transforming atrial electrograms recorded by a multi-electrode array to activation-phase signals and then quantifying the degree of similarity between snapshots of the activation-phase in the electrode array. An AF cycle length dependent distance threshold was applied to discriminate between repetitive and non-repetitive snapshots. Intervals containing repetitive conduction patterns were detected in a recurrence plot as regions with a high recurrence rate. Intervals that contained similar repetitive patterns were then grouped into clusters. To demonstrate the ability to detect and quantify the incidence, duration and size of repetitive patterns, the tool was applied to left and right atrial recordings in a goat model of different duration of persistent AF [3 weeks AF (3 wkAF, *n* = 8) and 22 weeks AF (22 wkAF, *n* = 8)], using a 249-electrode mapping array (2.4 mm inter-electrode distance).

**Results:**

Recurrence plots identified frequent recurrences of activation patterns in all recordings and indicated a strong correlation between recurrence plot threshold and AF cycle length. Prolonged AF duration was associated with shorter repetitive pattern duration [mean maximum duration 3 wkAF: 74 cycles, 95% confidence interval (54–94) vs. 22 wkAF: 41 cycles (21–62), *p* = 0.03], and smaller recurrent regions within repetitive patterns [3 wkAF 1.7 cm^2^ (1.0–2.3) vs. 22 wkAF 0.5 cm^2^ (0.0–1.2), *p* = 0.02]. Both breakthrough patterns and re-entry were identified as repetitive conduction patterns.

**Conclusion:**

Recurrence plots provide a novel way to delineate high-density contact mapping of AF. Dominant repetitive conduction patterns were identified in a goat model of sustained AF. Application of the developed methodology using the new generation of multi-electrode catheters could identify additional targets for catheter ablation of AF.

## Introduction

During atrial fibrillation (AF) electrical conduction patterns in the atria are divers, variable, and often complex. The complexity of these wave front patterns, i.e., the number of waves that propagate through the atria during each AF cycle, typically increases with AF duration (Allessie et al., 2010). Catheter ablation of AF aims at isolation of triggers for AF and at elimination of a dominant electrical mechanism that initiates or sustains AF. Current success rates of various approaches to catheter ablation of AF in patients show that there is a need to systematically identify additional targets besides the pulmonary veins (PV), especially in patients undergoing redo procedures after initially successful PV isolation. Several candidate mechanisms, associated detection algorithms, and ablation strategies have been proposed and applied in the last few decades, but this has not yet led to significantly improved long-term ablation outcome (Verma et al., 2015; Wong et al., 2015).

Proposed candidate sources of AF often show a high degree of repetitiveness. Repetitive focal patterns of activation detected in high-density mapping have been reported in patients with persistent AF (Holm et al., 1997; Lee et al., 2015), but others found repetitive focal events to be rare (de Groot and Allessie, 2019). Highly repetitive microreentrant sources were found using optical mapping, both in sheep (Mandapati et al., 2000) and in a small diverse set of human explanted hearts (Hansen et al., 2015), but also using panoramic contact mapping (Swarup et al., 2014). Other studies suggest more unstable spatiotemporal behavior of reentrant circuits, driven by an underlying stochastic process of wave generation (Dharmaprani et al., 2019) or clustered at the borders of fibrotic atrial regions (Haïssaguerre et al., 2016). Repetitive AF sources were also identified in several anatomical locations with a more general approach based on high electrogram morphology similarity and short AF cycle length (Ravelli et al., 2012, 2014).

Repetitive conduction is also to be expected in the vicinity of such local drivers. A local source may not always conduct 1:1 to its vicinity but this region is nonetheless expected to exhibit repetitive conduction patterns driven by the source. The presence of repetitive patterns in a region of the atria may furthermore reveal a structure-function relationship at that site, impacted by atrial anatomy (Hansen et al., 2015; van Hunnik et al., 2018) or by structural remodeling associated with AF (Verheule et al., 2010; Maesen et al., 2013). A repetitive pattern may also be the precursor to (spontaneous) termination of AF (Ortiz et al., 1993), or give an indication of the overall state of atrial conduction, i.e., the dynamics of linking of conduction between consecutive activations in different atrial regions (Gerstenfeld et al., 1992). Therefore techniques to reliably identify repetitive conduction patterns can be very instrumental, particularly in the light of recent advances toward electro-anatomical mapping tools with increased spatiotemporal resolution.

In this paper we introduce a method to analyze the incidence and characteristics of repetitive conduction patterns in contact mapping of AF using a recurrence plot, a well-established technique to study the dynamics of complex non-linear systems (Marwan et al., 2007). We demonstrate the ability of this novel computational tool to detect and quantify repetitive conduction patterns in high-density epicardial mapping in a goat model of AF.

## Materials and Methods

### High-Density Contact Mapping in a Goat Model of AF

In this study we made use of a retrospective dataset, comprised of baseline measurements from a drug provocation study in two groups of 8 goats, in which AF was induced by left atrial burst pacing and maintained for either 3 weeks (3 wkAF) or 22 weeks (22 wkAF). High-density contact mapping was performed during an open-chest experiment, using a 249-electrode circular mapping array (2.4 mm inter-electrode distance, sampling frequency 1039 Hz). One-minute recordings of unipolar atrial electrograms were made simultaneously on the right atrial (RA) and left atrial (LA) epicardial wall. Further experimental details can be found in van Hunnik et al. (2018). The study protocol was approved by the local ethics committee and complied with the Dutch and European directives.

### Recurrence Plot Construction to Visualize Repetitive Pattern Incidence

Recurrence plots provide a general way to visualize and analyze the temporal behavior of complex non-linear dynamical systems (Marwan et al., 2007). It requires the definition of a phase-space trajectory of the dynamical system and a distance function that measures the similarity or distance between any pair of time points on the trajectory. A recurrence then occurs when the distance between two points in time is below a certain threshold. The recurrence rate *RR* is defined as the fraction of detected recurrences over all comparisons.

**FIGURE 1:**
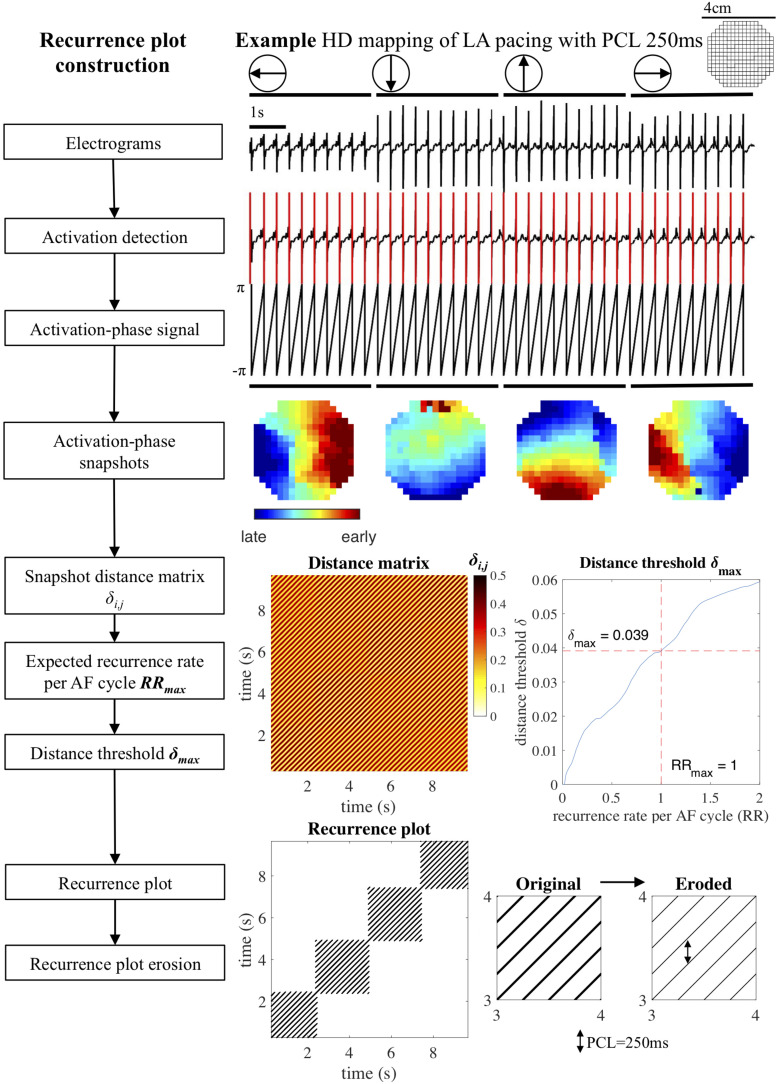
Recurrence plot construction. Block diagram and example of recurrence plot reconstruction for a recording of a paced rhythm. Unipolar electrograms were annotated, converted to activation-phase signals and used to create activation phase snapshots. Next, the distances δ_i,j_ between all pairs of snapshots (*i,j*) were used to construct a distance matrix. The distance threshold δ*_max_* was computed based on the maximum recurrence rate per AF cycle *RR*_max_ (default *RR*_max_ = 1) and applied to the distance matrix to construct a recurrence plot. Consecutive recurrences were eroded, removing false positives from the recurrence plot. HD, high density; LA, left atrium; PCL, pacing cycle length.

We adapted this general definition of recurrence plots to the analysis of atrial electrograms and conduction patterns. The approach is illustrated in Figure 1 on a recording of beats that were regularly paced from four cardinal directions. First, unipolar atrial electrograms were transformed to activation-phase signals by local activation time annotation, employing a previously published algorithm that assigns local atrial deflections to maximize the likelihood of atrial deflection intervals given an estimated AF cycle length distribution (Zeemering et al., 2012). Activation-phase signals (in the range [−π, π)] were constructed by linear interpolation, taking the local activation time as the moment of phase inversion. The phase-space was then defined as the snapshot of the activation-phase of all mapping array electrodes at individual time points. This snapshot can be interpreted as still frame of the local conduction pattern at a given time point. The distance δ_i,j_ between two time points *i* and *j* on the phase-space trajectory was determined based on the phase angle difference at every electrode, by taking the average of the cosine of each difference, transformed back to a fraction of the activation-phase duration of a single AF cycle (2π). This distance measure was chosen so that the range of differences between two snapshots [(−2π, 2π)] was mapped appropriately: maximum similarity occurred at differences (0, −2π, and 2π), while maximum dissimilarity occurred at π and −π. This also meant that the distance between two snapshots was always symmetric. The resulting distance measure ranged from 0 (two snapshots that were completely in phase) to 0.5 (two snapshots that were half an AF cycle length out of phase).

This transformation of electrograms to activation-phase snapshots, also known as the phase-space embedding, together with the distance function was subsequently used to create a distance matrix in which all activation-phase snapshots within a recording were compared. The distance matrix was transformed to a recurrence plot by imposing an adaptive maximum distance threshold δ*_max_*. The distance threshold δ*_max_* can be interpreted as the maximum degree to which two snapshots were allowed to be out of phase for a recurrence to occur. This threshold was computed by requiring the recurrence plot to have a recurrence rate that corresponded to a maximum recurrence rate per AF cycle *RR*_max_. Default value for *RR*_max_ was set to 1, equivalent to the recurrence rate per AF cycle that would occur if a single conduction pattern were repeating for the whole length of the recording. The resulting δ*_max_* and recurrence rate of the recurrence plot therefore depended on the AF cycle length, the number of time points in the recording, and the distribution of distances in the distance matrix. A formal definition of the distance matrix, recurrence plot construction, and distance threshold computation is provided in the section “Distance Matrix and Distance Threshold Computation” of Supplementary Methods. Note that this choice for the threshold constituted a sensitive detection of recurrences, which on the one hand ensured that completely regular patterns were detected correctly, but on the other hand also caused false positive recurrence detections (also known as false nearest neighbors in recurrence plot analysis) in recordings with a lower degree of regularity.

### Recurrence Plot Analysis to Detect Repetitive Patterns

Recurrence plots were analyzed to detect the incidence and duration of repetitive patterns. There are a few general features of a recurrence plot that are worth mentioning here. A recurrence plot is always symmetric as it compares all pairs of snapshot within a recording in both directions of time, past and future. The main diagonal represents the comparison of a snapshot with itself and hence is always recurrent. A repeating conduction pattern will form a sequence of consecutive recurrences, i.e., if two activation-phase snapshots are similar, these two snapshots will also be similar when shifted equal (small) amounts in time. This phenomenon is visible in a recurrence plot as a diagonal line (Marwan et al., 2007). A conduction pattern that repeats consistently for several consecutive AF cycles will show up in the recurrence plot as a square block of diagonal lines around the main diagonal. Due to the sensitive detection of recurrences, diagonal lines may exhibit some “thickness” when the distance between consecutive activation-phase snapshots still falls within the imposed threshold, which leads to aforementioned false positive recurrences. In our analysis this effect was removed by eroding the recurrence plot, effectively replacing consecutive horizontal or vertical snapshot recurrences by a single recurrence at the time point with minimum distance.

**FIGURE 2:**
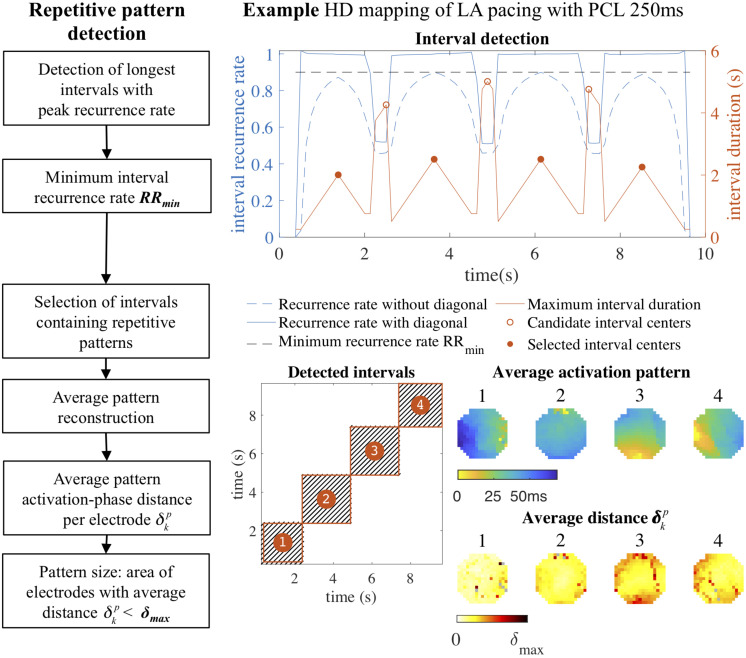
Repetitive pattern detection. Intervals containing repetitive patterns were detected by traversing the main diagonal of the recurrence plot and computing the duration of the square block surrounding the diagonal with the maximum recurrence rate at each time point. Intervals with peak duration and a recurrence rate above a minimum recurrence rate per AF cycle *RR*_min_ (0.9) were selected. In the four detected intervals the average activation time map was constructed, representing the original four pacing directions. For each pattern a heat map was constructed that indicated the average recurrent activation-phase distance $\delta_{\text{k}}^{\text{p}}$ for a pattern (*p*) for each electrode (*k*) separately. From this heat map the pattern size was computed by determining the area of the electrodes with $\delta_{\text{k}}^{\text{p}}<\delta_{\text{max}}$. In this example of a paced rhythm the repetitive patterns were very consistent: almost all electrodes contributed to the repetitive pattern with an average activation-phase distance below the distance threshold. HD, high density; LA, left atrium; PCL, pacing cycle length.

The resulting eroded recurrence plot was then used to detect intervals that contained repetitive patterns, as illustrated in Figure 2. These intervals were detected by an algorithm that traversed the main diagonal of a recurrence plot and computed the recurrence rate per AF cycle in square blocks of increasing duration around the diagonal at each time point. Recurrence rate was again normalized to the AF cycle length, so that a value of 1 indicated a single recurrence occurring each AF cycle on average. For each time point we stored the duration of the interval that was formed by the square block in the recurrence plot with the maximum recurrence rate. Intervals that contained repetitive patterns were defined as local maxima in the interval duration, with a minimum recurrence rate per AF cycle *RR*_min_. The default value of *RR*_min_ was set to 0.9, corresponding to an average of 0.9 recurrences per AF cycle, to account for short-lasting pattern interruptions and small deviations in AF cycle length. In case of two identified intervals with more than 50% overlap in time, the interval with the longest duration was selected.

### Clustering of Similar Repetitive Patterns

To investigate which identified intervals on the main diagonal of the recurrence plot containing repetitive patterns represented similar or distinct activation patterns, patterns were grouped based on the cross-recurrence rate between intervals. Cross-recurrence rate between two intervals was determined by computing the recurrence rate in the rectangular block in the recurrence plot formed by the respective intervals. The resulting interval similarity matrix, describing the cross-recurrence rate between all pairs of intervals containing repetitive patterns, was clustered using agglomerative hierarchical clustering, a commonly used clustering technique that builds a tree of linked pairs of most similar intervals using a bottom-up approach (Hastie et al., 2009). Groups of similar patterns were extracted from the hierarchical cluster tree using the same recurrence rate threshold *RR*_min_ applied in the detection of repetitive patterns.

### Repetitive Pattern Visualization

Repetitive (clustered) patterns were visualized by computing the average of activation-phase snapshots corresponding to recurrences on a vertical line within the corresponding intervals in the recurrence plot. The average activation-phase snapshot was converted to an average activation time map using the estimated AF cycle length, setting the earliest activation time to zero. To identify spatial regions within the mapping array that contributed most to the recurrent behavior of the average pattern, a heat map was constructed that indicated the average recurrent activation-phase distance $\delta_{\text{k}}^{\text{p}}$ for a pattern (*p*) for each electrode (*k*) separately (see section “Average Pattern Activation-Phase Distance” in Supplementary Methods). The size of the most recurrent region was defined as the area covered by electrodes with an average activation-phase distance below the computed adaptive distance threshold δ*_max_*, or a fixed time difference threshold Δ*t*, after converting the average activation-phase distance per electrode to a time difference by multiplying $\delta_{\text{k}}^{\text{p}}$ by the AF cycle length.

### Statistics

Based on the recurrence plot analysis we determined for each individual recording the adaptive distance threshold δ*_max_*, the number and duration of intervals containing repetitive patterns and clusters of similar patterns, and the size of most recurrent region. Sensitivity analysis was performed to assess the effect of the thresholds *RR*_max_ and *RR*_min_ on the detection of repetitive patterns (see section “Sensitivity Analysis” in Supplementary Methods). Differences between 3 and 22 wkAF and LA and RA were tested using mixed ANOVA, employing a significance threshold of 0.05. Correlations between parameters were computed using Spearman’s rank correlation coefficient, controlling for AF group and atrium. Algorithms for recurrence plot construction and repetitive pattern detection were implemented in MATLAB (2019). Statistical tests were performed using R (R Core Team, 2019) and the package *emmeans* (Lenth, 2020).

## Results

### Application to High-Density Recordings of AF

**FIGURE 3:**
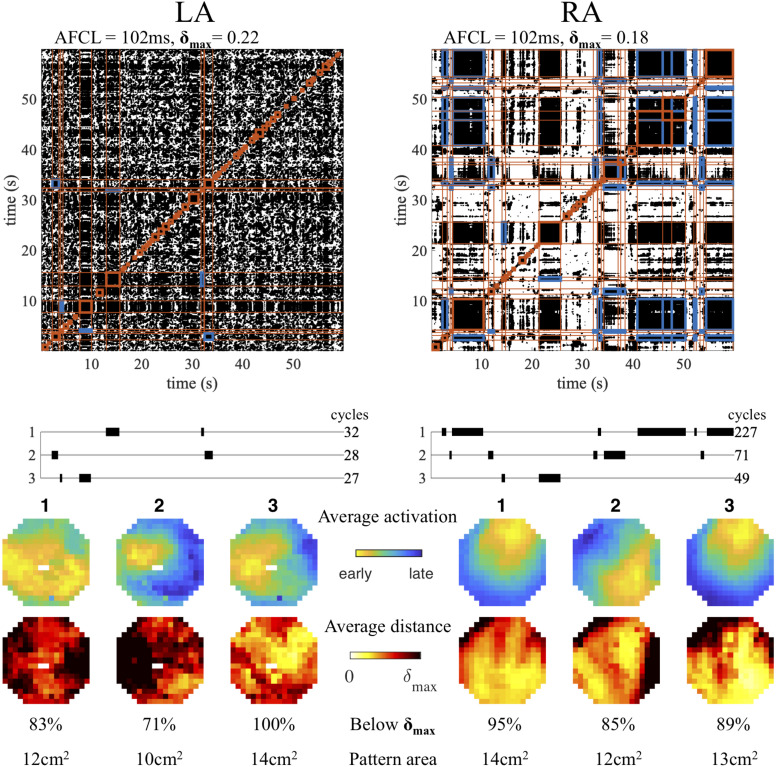
Example of repetitive pattern detection in simultaneous **left** (LA) and **right** (RA) atrial recordings. For both locations the recurrence plot and interval detection (red blocks) are depicted. Those intervals are shown that belong to the three clustered patterns with longest duration (in cycles). Cross-recurrence between intervals belonging to the same cluster is indicated in blue in the recurrence plot. The average activation pattern for each pattern is illustrated, together with the heat map of the average activation-phase distance per electrode.

The procedure of electrogram activation-phase and snapshot distance matrix computation, recurrence plot construction and repetitive pattern detection was applied to recordings during AF in the goat model. The result of this automated analysis scheme in simultaneous left and right atrial recordings in a single animal is illustrated in Figure 3. Intervals containing repetitive patterns are indicated as red square blocks around the diagonal. For both atria, the detected intervals were clustered into groups of similar patterns, of which the three clusters with the longest combined duration (in AF cycles) are shown. For each clustered pattern the average activation time map was determined together with the heat map of the average activation-phase distance per electrode. In this case the right atrium showed a repetitive pattern for many cycles (227), a wave entering from the northeast of the mapping area, alternated with a different pattern (71 cycles), again a wave entering the field of view, but now from the southeast. The third pattern resembled the first, but the distance heat map indicated a more variable pattern at the entrance point of the wave. In contrast, at the same time in the left atrium, the three most prevalent patterns were short lasting, with 32, 28, and 27 cycles, respectively. Here the first pattern represented two peripheral waves that collided in the center of the mapping area, and the second and third repetitive focal/breakthrough waves at distinct locations. The heat maps of the patterns in the left atrium indicated a more variable or unstable pattern compared to the right.

### Sensitivity Analysis of *RR*_max_ and *RR*_min_

First, we varied *RR*_max_ between 0.25 and 2, around the default value of 1, and quantified the effect on the recurrence plot construction: the distance threshold δ*_max_* and the recurrence rate of the eroded recurrence plot. We also evaluated the effect of *RR*_max_ on the detection of repetitive patterns (keeping *RR*_min_ fixed at the default value 0.9): number of intervals, maximum duration of an interval, maximum pattern duration and pattern size (using the adaptive distance threshold δ*_max_* that is determined by the choice of *RR*_max_). We repeated this analysis for a range of fixed δ*_max_*. Results show that most parameters are moderately affected by changes in *RR*_max_. Recurrence plot parameters show an approximately linear response (Supplementary Figures 1A,B), while the parameters related to the detection of repetitive patterns show either a linear (maximum interval and pattern duration), a weak biphasic (number of intervals), or almost no response (pattern size) (Supplementary Figure 2). In contrast, using a range of fixed δ*_max_* [0.1, 0.25] that corresponded to the range of observed values for the δ*_max_* computed from *RR*_max_, we observed a much stronger, non-linear response in the eroded recurrence plot recurrence rate (Supplementary Figure 1C) and the pattern detection parameters (Supplementary Figure 3). Supplementary Figure 4 provides an example of recurrence plot construction and repetitive pattern detection for varying values of *RR*_max_.

Second, we varied *RR*_min_ between 0.5 and 1.5, while keeping *RR*_max_ fixed at *RR*_max_ = 1. Results show that choices for *RR*_min_ close to the default value 0.9 do not change the qualitative interpretation of the results of the pattern identification, most notably for the maximum duration of patterns and the pattern size (Supplementary Figure 5).

### Repetitive Patterns in a Goat Model of Different AF Duration

**FIGURE 4:**
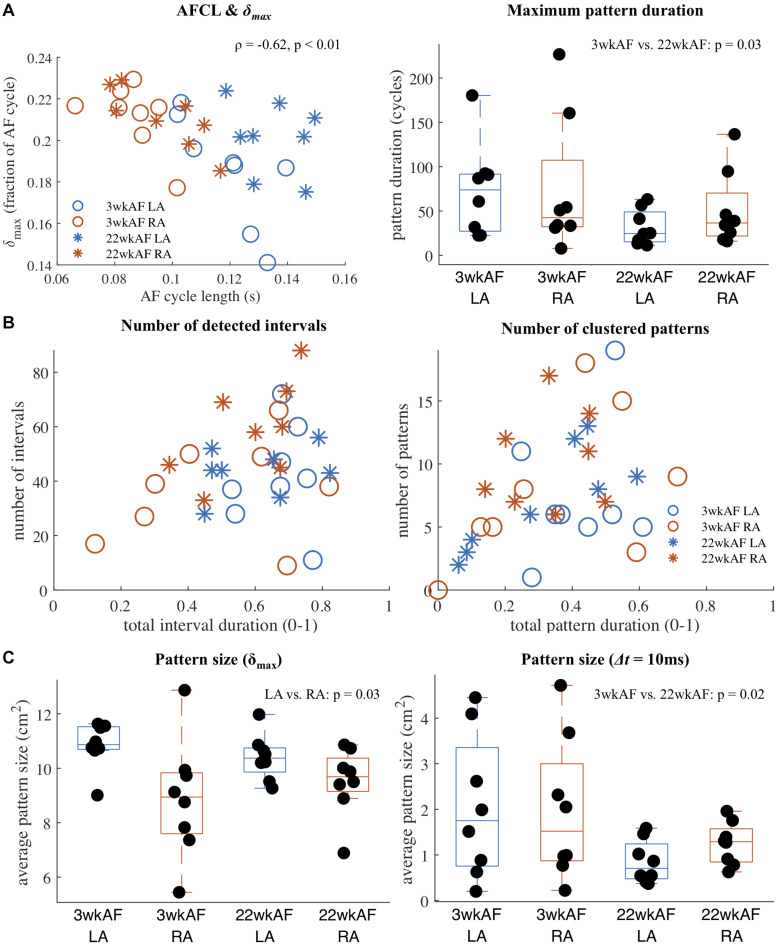
Recurrence detection results in 3 and 22 wkAF goats. **(A)** Correlation between AF cycle length (AFCL) and the adaptive distance threshold δ*_max_*, and maximum pattern duration per recording in AF cycles. **(B)** Variety of number of detected intervals containing a repetitive interval and clustered patterns compared to the total recording coverage. Only clustered patterns with a combined duration exceeding 10 AF cycles were included. **(C)** Average size of the most recurrent pattern region within each recording, computed using the adaptive distance threshold δ*_max_* (left) and a fixed maximum time difference Δ*t* (10 ms) applied to the average activation time difference between pattern recurrences (right).

Using *RR*_max_ = 1 and *RR*_min_ = 0.9, repetitive pattern detection was performed in all recordings at baseline in the two groups of 3 and 22 wkAF goats to investigate the incidence of repetitive patterns and to detect any potential differences in recurrence characteristics associated with AF duration and atrium. Our main results are summarized in Figure 4. The computed distance threshold δ*_max_* was strongly correlated with the AF cycle length (correlation −0.62, *p* < 0.01, Figure 4A). The maximum duration (in number of AF cycles) of clustered patterns was longer in 3 wkAF than in 22 wkAF (mean maximum duration 74 cycles [95% confidence interval (54–94) vs. 41 (21–62) cycles, *p* = 0.03]. In Figure 4B we illustrated the diversity in the number of intervals that contained repetitive patterns, as well as the total duration of these intervals during a recording. We observed a wide variety of interval incidence and prevalence, from recordings that showed a low number of intervals that covered only a small portion of the recording duration, to recordings with a small to large amount of intervals that covered almost the entire recording. The number of patterns, where multiple intervals could be grouped into one clusters representing a single pattern, compared to the total coverage of the recording showed similar diversity. Here we only included clusters of patterns where the total duration of the combined intervals exceeded 10 AF cycles. The average size of the most recurrent region per recording (Figure 4C) was slightly smaller in the right atrium [mean size LA 10.9 cm^2^ (10.0–11.4) vs. RA 9.5 cm^2^ (8.8–10.3), *p* = 0.03], when computed based on the number of electrodes with an average activation-phase distance below the adaptive distance threshold δ*_max_*. Pattern region size was much smaller when applying a fixed maximum activation time difference threshold Δ*t* of 10 ms between recurrences in detected patterns. Here, differences were found between 3 and 22 wkAF [3 wkAF 1.7 cm^2^ (1.0–2.3) vs. 22 wkAF 0.5 cm^2^ (0.0–1.2), *p* = 0.02]. Sensitivity analysis of Δ*t* indicated that this difference in regions with low average temporal dissociation was consistent for values of Δ*t* between 10 and 30 ms (Supplementary Figure 5B).

### Examples of Mechanisms Detected by Recurrence Analysis

**FIGURE 5:**
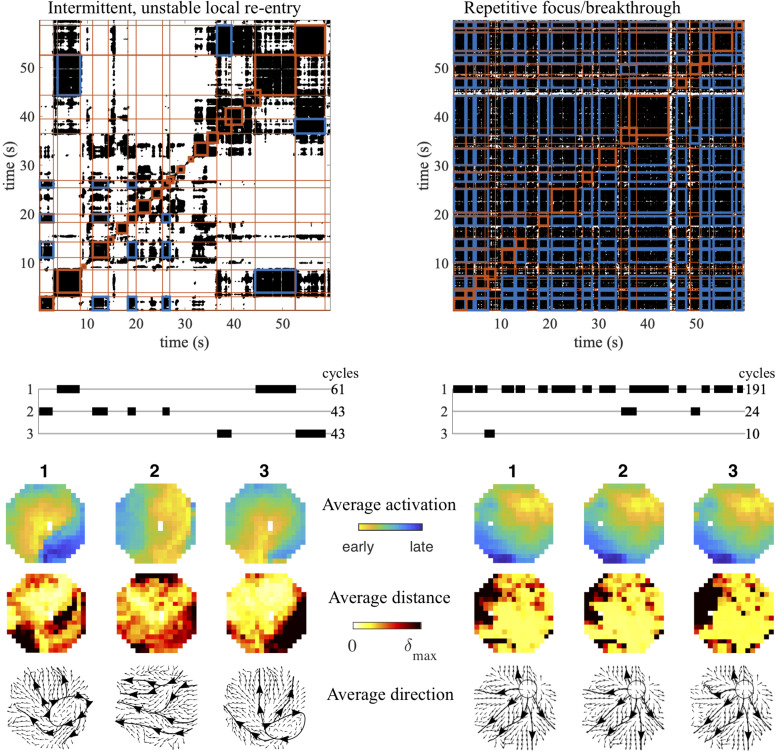
Examples of AF mechanisms detected using recurrence analysis. Two conduction patterns associated with candidate AF mechanisms are depicted: an intermittent, unstable local re-entry within the mapping area **(left)** and a repetitive focal/breakthrough wave **(right)**. Intervals containing recurrent patterns are indicated in red blocks, while cross-recurrences between clustered intervals are indicated in blue. The average activation pattern for each pattern is illustrated, together with the heat map of the average activation-phase distance per electrode and the average conduction direction during the pattern duration.

In Figure 5 we show two examples to illustrate how this method can detect and visualize candidate AF source patterns. These were selected recordings from the same study in goats, but during infusion of different dosages of the antiarrhythmic drug used. The first example shows a recording that started with a peripheral wave entering from the east (pattern 2), but then switched to a local re-entry within the mapping area (pattern 1). Pattern 2 returned after a while, but was intermitted by other, less stable and frequent patterns. Then pattern 3 arose, again a local re-entry within the mapping area, comparable to pattern 1, but following a slightly different trajectory. Finally pattern 1 reappeared, followed by pattern 3. The dominant conduction direction per electrode for each of the patterns confirmed their interpretation. The second example shows an extreme case of a repetitive focal/breakthrough wave, where the most spatiotemporal stable pattern (pattern 1) appeared intermittently for a total of 191 cycles. In this example the other intermittent patterns (patterns 2 and 3) were very similar to pattern 1, and only differed slightly in the variation of the radial spread of activation, as indicated by the activation-phase distance heat maps. The dominant conduction direction per electrode for each of the patterns highlights this radial spread originating from the site of the focus/breakthrough activation.

## Discussion

We developed a method to automatically identify repetitive patterns of conduction in high-density mapping of AF. This method can be used to detect the incidence of time intervals containing repetitive patterns, to group intermittent intervals that exhibit similar repetitive patterns, and to visualize these distinct patterns to interpret the mechanism the conduction pattern represents. As an application, repetitive conduction patterns were identified in HD mapping recordings in a goat model of sustained AF, where we identified repetitive patterns in almost all recordings. We also show that the maximum duration of repetitive patterns and the size of the regions containing the most dominant repetitive pattern decreased with prolonged AF duration.

### Recurrence Analysis as a Tool to Identify Repetitive Patterns During AF

Recurrence analysis has been applied to investigate AF characteristics in invasive measurements, based on single electrode bipolar electrogram morphology. Recurrence plots were used to assess the degree of organization present during AF (Censi et al., 2000). Recurrence quantification analysis was applied to detect complex fractionated atrial electrograms (Navoret et al., 2013), to quantify the dynamics of beat-to-beat deflection morphology similarity at several locations in the left and right atrium (Ng et al., 2014), and to distinguish spiral wave reentry from multiple wavelets in bipolar electrograms (Hummel et al., 2017). Recurrence plots derived from consecutive AF cycle lengths from an electrogram recorded in the coronary sinus suggested that the underlying AF process is deterministic, rather than stochastic (Aronis et al., 2018). Also alternative methods for the detection of local bipolar electrogram regularity were proposed, again using electrogram morphology, but also by integrating electrogram coupling to quantify local organization (Faes and Ravelli, 2007), or by quantifying the repetitiveness of the pattern of complex fractionated atrial electrograms using frequency domain parameters (Ciaccio et al., 2011). The use of a single electrode and bipolar electrograms, however, limits the amount of spatiotemporal information that can be incorporated in the assessment of the dynamics of the underlying recurrent conduction pattern. In our approach we aimed to not only include temporal dynamics, but also detailed local spatial coherence, by analyzing electrograms from a high-resolution grid of electrodes. Our approach further relies on unipolar electrograms that are insensitive to the direction of conduction. We adopted an activation-phase representation of the electrograms to enable the computation of the distance, or difference, between two snapshots of conduction in time. Other approaches may also be chosen to arrive at this phase-space representation of the conduction patterns, for instance methods that rely on the Hilbert transform of the filtered electrogram (Kuklik et al., 2015). Caution should be applied, however, when interpreting the resulting averaged activation-phase patterns, as these approaches tend to blur the true underlying activation patterns, which can lead to the elimination of conduction block (Podziemski et al., 2018).

### Interpretation and Implications of the Adaptive Recurrence Threshold

In our approach we adopted an adaptive distance threshold to transform the distance matrix that describes the difference in activation-phase between each pair of snapshots, to a recurrence plot, that only contains the moments in time when two conduction patterns are sufficiently in phase. The choice for a recurrence plot threshold has to be made carefully: applying a too restrictive threshold will not identify existing recurrences and can lead to the detection of many short, interrupted intervals; a too tolerant threshold will lead to many false positive recurrences, that obscure the underlying structure of the recurrence plot (Marwan, 2011). There are several approaches to choosing a recurrence threshold (Zbilut et al., 2002). We used the *a priori* knowledge of the AF cycle length to choose a threshold that led to a number of recurrences that was to be expected if the underlying pattern was completely regular and repetitive for the whole duration of the recording. In the case of AF this threshold was often too tolerant, which led to sensitive, but not specific detection of recurrent snapshots. The post processing of the resulting recurrence plot, together with the constraints imposed on the detection of repetitive patterns, ensured that these false positives detections were disregarded. Sensitivity analysis indicated that this approach to compute an adaptive distance threshold is a relatively robust choice compared to setting a fixed distance threshold: a small change in *RR*_max_ led to relatively small and predictable change in δ*_max_* and associated pattern detection results, whereas changes in a fixed δ*_max_*, that was applied to all recordings, led to much more pronounced and unpredictable changes in pattern detection results.

A higher recurrence threshold that still leads to the detection of intervals that contain repetitive intervals can indicate two things: either repetitive patterns are more variable, but still stand out from other intervals with even more disorganized activity, and/or the spatial region within the mapping area where the repetitive pattern is localized is smaller. The application of our method on the goat model data revealed that the threshold is strongly associated with the AF cycle length. This correlation indicated less stable or smaller repetitive patterns in recordings with shorted AF cycle lengths, which corroborates the findings of Schuessler et al. (1992) where in a cholinergic model of AF the shortening of the effective refractory period (and cycle length) resulted in an increased number of wave fronts and local re-entry circuits.

### Repetitive Patterns in AF

Applying the developed methodology to recordings in a goat model of AF, we found that there was a large diversity both in the number of repetitive patterns as well as in the total duration of the recording covered by repetitive patterns. Stability of patterns, however, seemed to decrease with AF duration, with a lower maximum pattern duration in 22 wkAF. The size of the region within the mapping area responsible for the recurrent behavior did also decrease with AF duration (using a fixed threshold of 10ms for the maximum allowed activation time difference between recurrences). This suggests that, while repetitive patterns are still present, the size of the repetitive process becomes smaller with prolonged AF duration. This is largely in line with findings in a comparable goat model of AF (Verheule et al., 2010). Interestingly we observed switching between different repetitive patterns in several of the examples (Figures 3, 5). This suggests the existence of different states of the atrial conduction during AF, and sudden transitions between these states, as also observed in simulations of AF (Iravanian and Langberg, 2017; Marcotte and Grigoriev, 2017).

In this study we analyzed mapping data from the epicardium of both atria. Since conduction during AF is a 3D process, signals measured simultaneously on the endocardial wall may show some degree of uncoupling (Eckstein et al., 2013; Gutbrod et al., 2015; de Groot et al., 2016). Independent epi-endocardial repetitive patterns might point to separate drivers in the two layers. It would be of great interest to further investigated whether epi-endocardial coupling occurs during episodes of repetitive activity. Furthermore, electrode array resolution has been shown to significantly impact AF driver identification (Roney et al., 2017). Computation modeling of AF can help to investigate the effect of 3D conduction, dissociation and mapping device on pattern detection, providing that the model incorporates the 3D nature of the atrial anatomy and bundle structure, and exhibits epi-endocardial dissociation to an extent that 3D conduction patterns can be simulated [see for instance (Gharaviri et al., 2020)].

### Recurrence Analysis to Identify and Target AF Sources

The detection of AF sources during an AF ablation is a possible extension of the current approach. The examples in Figure 5 show that our approach – in principle – can detect and visualize local candidate mechanism that may drive or initiate AF (e.g., a local re-entry or a repetitive focal/breakthrough wave). In patients, ablation of such driver sites may restore sinus rhythm or prevent AF recurrence. As a future perspective, during an ablation procedure, several regions of the atria could be mapped sequentially and repetitive patterns can then be reconstructed for each region. With the use of a common reference, or spatial overlap between asynchronous recordings at different sites, repetitive patterns can be “stitched” together to form a more complete picture of whole atrial repetitive conduction. A similar approach was recently demonstrated in the RADAR trial (Choudry et al., 2020), where a catheter placed in the coronary sinus (CS) served as the common reference. A potential limitation of that specific setup is that intervals containing repetitive patterns were detected using only the electrograms from the CS catheter. In contrast, identification of repetitive patterns at partially overlapping sites also enables the detection of candidate AF drivers that do not lead to repetitive electrogram morphology in the CS. Multi-site identification of repetitive patterns will, however, require recordings with longer duration than currently acquired during an ablation procedure, together with a sufficient incidence and duration of repetitive patterns.

## Limitations

The result of the recurrence plot construction and repetitive pattern detection were dependent on the chosen phase-space embedding (local activation-phase computation) and recurrence rate thresholds *RR*_max_ and *RR*_min_. Optimal embedding and threshold values were not investigated in this study. Sensitivity analysis of *RR*_max_ and *RR*_min_ indicated that results obtained in the goat model of AF were moderately insensitive with respect to the exact choice for these thresholds. Recordings evaluated in this study were from a goat model of AF, where although AF was persistent, the amount of structural remodeling was most likely limited and the differences between groups subtle. A similar study in patients in different stages of AF (paroxysmal and persistent) is needed to investigate the relationship between AF duration and associated structural remodeling and repetitive pattern incidence and size.

## Data Availability Statement

The raw data supporting the conclusions of this article will be made available by the authors, without undue reservation. Requests to access these datasets should be directed to SZ, s.zeemering@maastrichtuniversity.nl.

## Ethics Statement

The animal study was reviewed and approved by the Local Ethical Board for animal experimentation of Maastricht University.

## Author Contributions

SZ developed the concept, developed analysis tools, analyzed and interpreted the data, and wrote the article. AH conducted the experiments, analyzed the data, and contributed to the scientific interpretation and the writing of the article. FR and PB developed analysis tools and contributed to the writing of the article. BS conducted the experiments. TD contributed to the scientific interpretation and the writing of the article. SV contributed to the scientific interpretation and the writing of the article. US developed the concept and contributed to the scientific interpretation and the writing of the article. All authors contributed to the article and approved the submitted version.

## Conflict of Interest

US is co-founder and shareholder of YourRhythmics BV, a spin-off company of the University Maastricht, holds intellectual property with Roche and YourRhythmics BV, received consultancy fees or honoraria from Johnson & Johnson, Roche Diagnostics (Switzerland), and Bayer Healthcare (Germany). The remaining authors declare that the research was conducted in the absence of any commercial or financial relationships that could be construed as a potential conflict of interest.
