# Supplementary material for: A Novel Tool for the Identification and Characterization of Repetitive Patterns in High-Density Contact Mapping of Atrial Fibrillation
Source: Front Physiol. 2020 Oct 15;11:570118. doi: 10.3389/fphys.2020.570118 (PMC7593698; doi:10.3389/fphys.2020.570118)
Supplement: Supplementary file 1 [file Data_Sheet_1.PDF]

Supplemental material

Supplemental methods

Distance matrix and distance threshold computation

The *distance* $\delta_{i,j}$ between two activation-phase snapshots at time points *i* and *j* was determined based on the phase angle difference at each of the *N* electrodes, by taking the average of the cosine of each difference, transformed back to a fraction of the activation-phase duration of a single AF cycle (2π). Formally:

$$\delta_{i,j} = \cos^{-1}\left(\sum_{k=1}^{N} \cos(\varphi_{k,j} - \varphi_{k,i}) \Big/ N\right) \Big/ 2\pi,$$

where $\varphi_{k,i}$ denotes the activation-phase of the electrode *k* at time *i*, and *N* the number of electrodes. This definition leads to distance values in the range [0, 0.5]. The distance matrix was then formed by computing $\delta_{i,j}$ for all combinations of *i* and *j*, where $i, j = 1,2, \dots, T$, with *T* being the total number of time points.

Recurrence $R_{i,j}$ between two snapshots was computed by imposing a distance threshold δ on the distance matrix:

$$R_{i,j} = \begin{cases} 0 \text{ if } \delta_{i,j} > \delta \\ 1 \text{ if } \delta_{i,j} \le \delta \end{cases}$$

The *recurrence rate* of a recurrence plot is defined as:

$$RR = \frac{1}{T^2} \sum_{i=1}^{T} \sum_{j=1}^{T} R_{i,j},$$

We adapted this definition to the *recurrence rate per AF cycle*, computed as the relative number of recurrences per AF cycle over all possible time point comparisons:

$$RR = \frac{T_{AFC}}{T^2}\sum_{i=1}^{T}\sum_{j=1}^{T} R_{i,j}\,,$$

where T_{AFC} denotes the number of time points per AF cycle.

The *distance threshold* δ_{max} applied to construct the recurrence plot for each recording was computed by selecting the largest value for the threshold δ that led to a given maximum recurrence rate per AF cycle RR_{max}:

$$\delta_{max} = \sup\{\delta \in [0, 0.5] \mid RR \leq RR_{max}\}.$$

Since

$$\sum_{i=1}^{T}\sum_{j=1}^{T} R_{i,j} = \frac{T^2}{T_{AFC}} RR_{max},$$

this is equivalent to computing the percentile of all T^2 distances in the distance matrix that corresponds to the expected recurrence rate RR_{max}, adjusted for the number of time points per AF cycle T_{AFC}:

$$\delta_{max} = P_{100\times RR_{max}/T_{AFC}}\{\delta_{i,j} \mid i,j = 1, \ldots, T\}.$$

Average pattern activation-phase distance

The average activation-phase distance for each electrode during a repetitive pattern was computed as the average distance at recurrences $R_{i,j}$ occurring during a repetitive pattern. Formally, given a pattern p and a corresponding set of time points T_p during which the pattern is detected, the average distance for an electrode k is given by:

$$\delta_k^p = \cos^{-1}\left(\sum_{i\in T_p}\sum_{j\in T_p} \cos(\varphi_{k,j} - \varphi_{k,i})\, R_{i,j} \Big/ \sum_{i\in T_p}\sum_{j\in T_p} R_{i,j}\right)\Big/ 2\pi$$

Sensitivity analysis

In this study, results were dependent on two main thresholds: 1) the maximum allowed distance between two activation-phase snapshots (δ_{max}) to construct a recurrence plot from a distance matrix, and 2) the minimal recurrence rate per AF cycle (RR_{min}) of an interval within the recurrence plot for which an interval was assumed to contain a repetitive pattern. The threshold δ_{max}, the recurrence plot threshold, was determined in a data-driven way for each recording separately, by computing the value of δ_{max} for which recurrence rate of the recurrence plot corresponded to the maximum recurrence rate per AF cycle (RR_{max}). Default values for RR_{max} and RR_{min} were 1 and 0.9 respectively. To assess the sensitivity of the results to the value of these thresholds, we performed recurrence plot construction and repetitive pattern detection for a range of RR_{max} and RR_{min} around the default values. To motivate the choice for a data-driven recurrence plot threshold δ_{max}, as opposed to an equal, fixed δ_{max} for all recordings, we also performed sensitivity analyses for a range of fixed δ_{max} values. To investigate the use of a fixed time threshold Δt when computing average pattern size, we performed sensitivity analysis on the maximum allowed average time difference per electrode during recurrences within a repetitive activation pattern. In this analysis, the average activation-phase distance for each electrode during a repetitive pattern δ_k^p was converted to time in milliseconds, by multiplying by the estimated AF cycle length of each recording.

Supplemental Figure legends

Supplemental Figure 1: Panel A: Effect of the maximum recurrence rate (RR_{max}) on the average of the maximum allowed distance between two activation-phase snapshots (δ_{max}) and change compared to δ_{max} at the default value $RR_{max} = 1$. Panel B: Effect of RR_{max} on the recurrence rate (RR) per AF cycle of the eroded recurrence plot

and change compared to RR at the default value RR_{max} = 1. Panel C: Effect of a fixed δ_{max} on the RR of the eroded recurrence plot and difference compared to RR when computed using δ_{max} at the default value RR_{max} = 1.

Supplemental Figure 2: Effect of the maximum recurrence rate per AF cycle (RR_{max}) on the detection of repetitive patterns. The minimal recurrence rate per AF cycle (RR_{min}) was held constant in this analysis at RR_{min} = 0.9.

Supplemental Figure 3: Effect of a fixed distance threshold δ_{max} for all recordings to construct a recurrence plot on the detection of repetitive patterns. The minimal recurrence rate per AF cycle (RR_{min}) was held constant in this analysis at RR_{min} = 0.9.

Supplemental Figure 4: Example of the effect of various values for the maximum recurrence rate (RR_{max}) on the recurrence plot construction and interval and pattern detection in a single recording. Upper panel: recurrence plots, interval detection (red blocks) and cross-recurrence between intervals belonging to the same cluster (blue blocks), for RR_{max} = 0.50, 1, 1.5 and 2. Lower panel: *left*: RR_{max} -driven change in δ_{max} and recurrence rate (RR) of the eroded recurrence plot (RP); *middle*: the number of intervals containing repetitive patterns and maximum interval duration; *right*: the number of clustered patterns and pattern size (area formed by electrodes with an average activation-phase distance during pattern recurrences below δ_{max}). The minimal recurrence rate per AF cycle (RR_{min}) was held constant in this analysis at RR_{min} = 0.9.

Supplemental Figure 5: Panel A: Effect of the minimum recurrence rate per AF cycle of an interval containing a repetitive pattern (RR_{min}) on the detection of repetitive patterns. The maximal recurrence rate per AF cycle (RR_{max}) was held constant in this analysis at $RR_{max} = 1$. Panel B: Effect of a fixed threshold Δt in milliseconds to compute the size of repetitive patterns. Here the average activation-phase distance for each electrode during a repetitive pattern δ_k^p was converted to time in milliseconds, by multiplying δ_k^p by the estimated AF cycle length of each recording. Repetitive patterns were computed using $RR_{max}=1$ and $RR_{min} = 0.9$.
