# Supplementary figures and images for: A Novel Tool for the Identification and Characterization of Repetitive Patterns in High-Density Contact Mapping of Atrial Fibrillation
Source: Front Physiol. 2020 Oct 15;11:570118. doi: 10.3389/fphys.2020.570118 (PMC7593698; doi:10.3389/fphys.2020.570118)

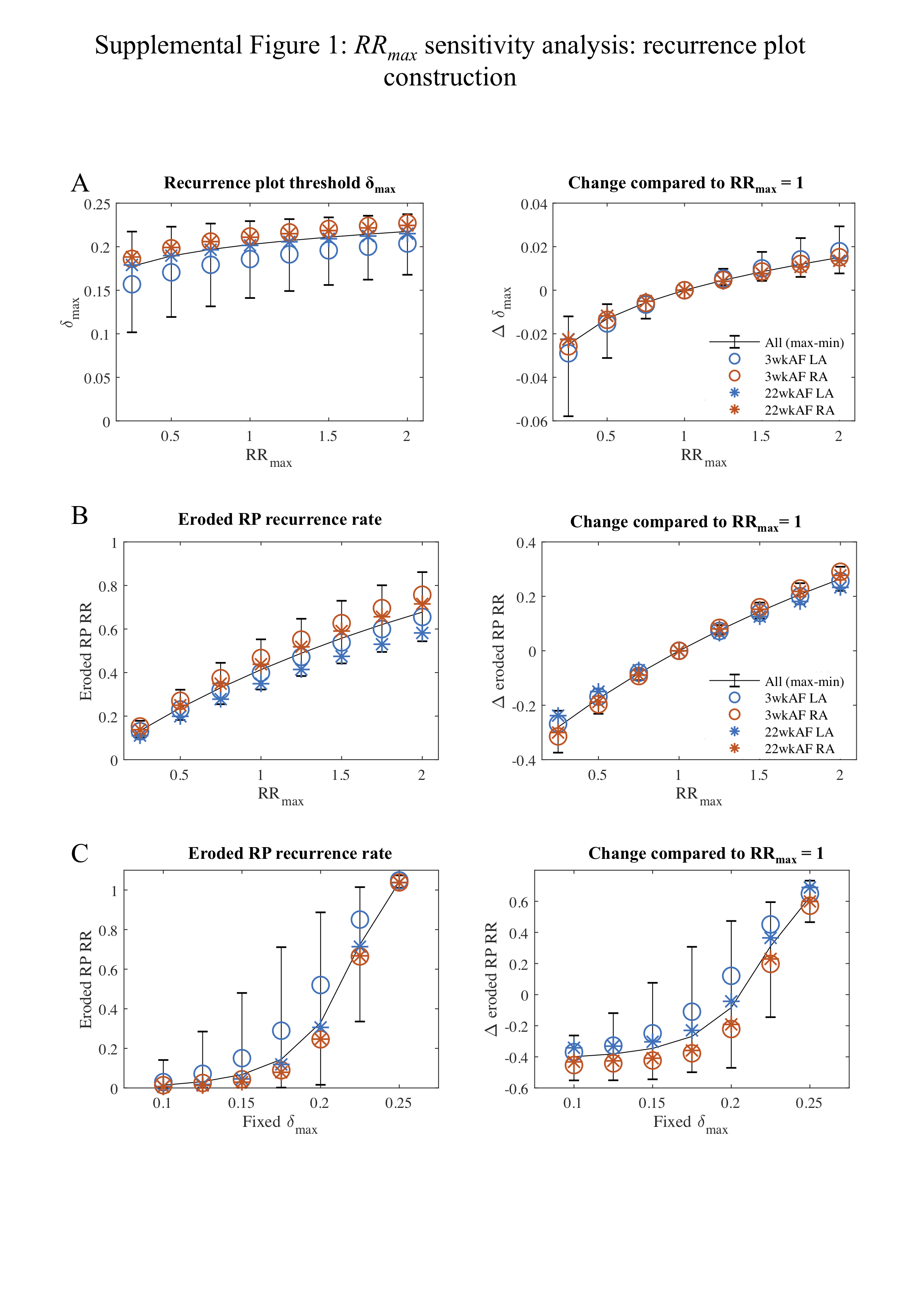

Supplement: Supplementary file 2 [file Image_1.JPEG]

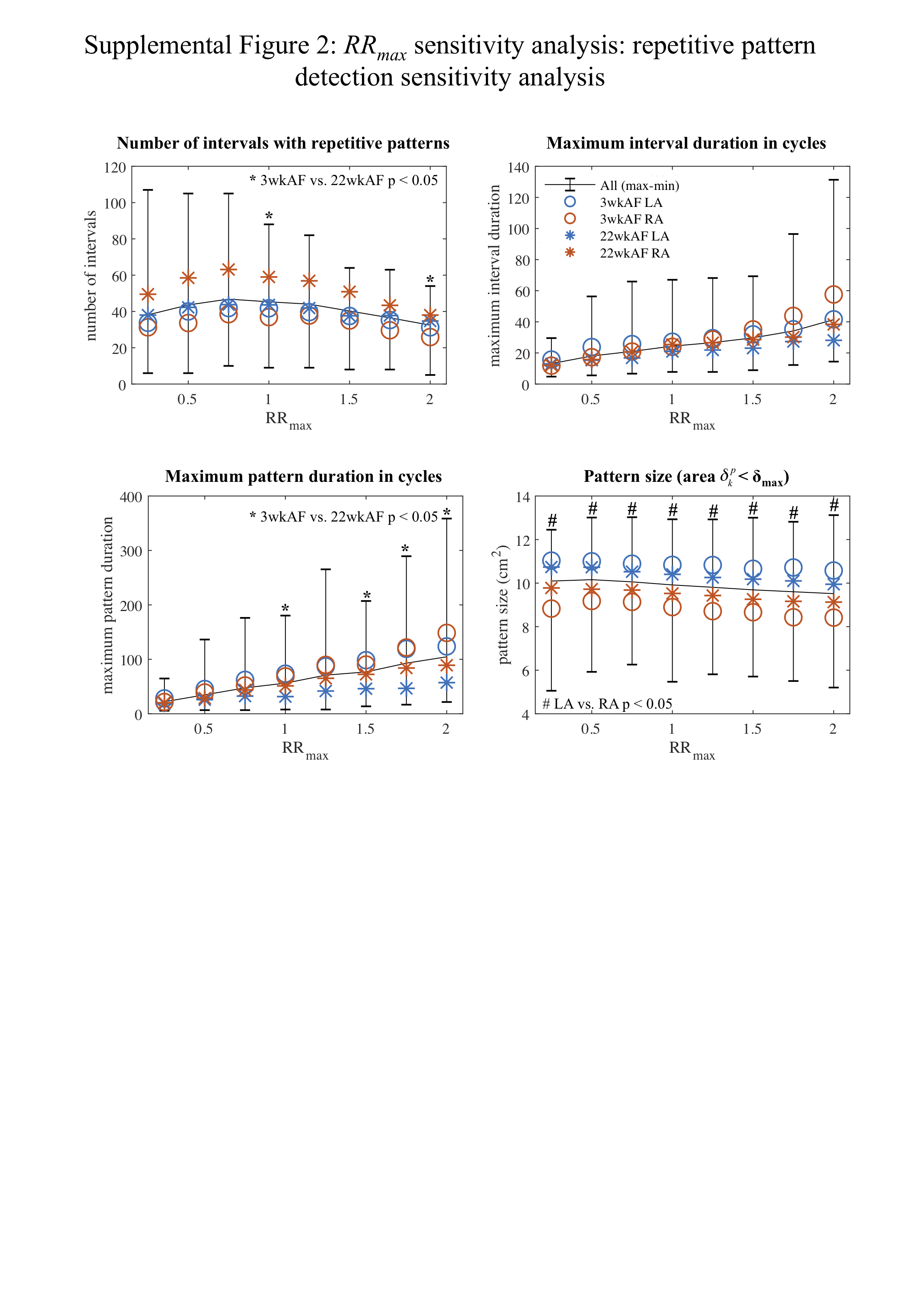

Supplement: Supplementary file 3 [file Image_2.JPEG]

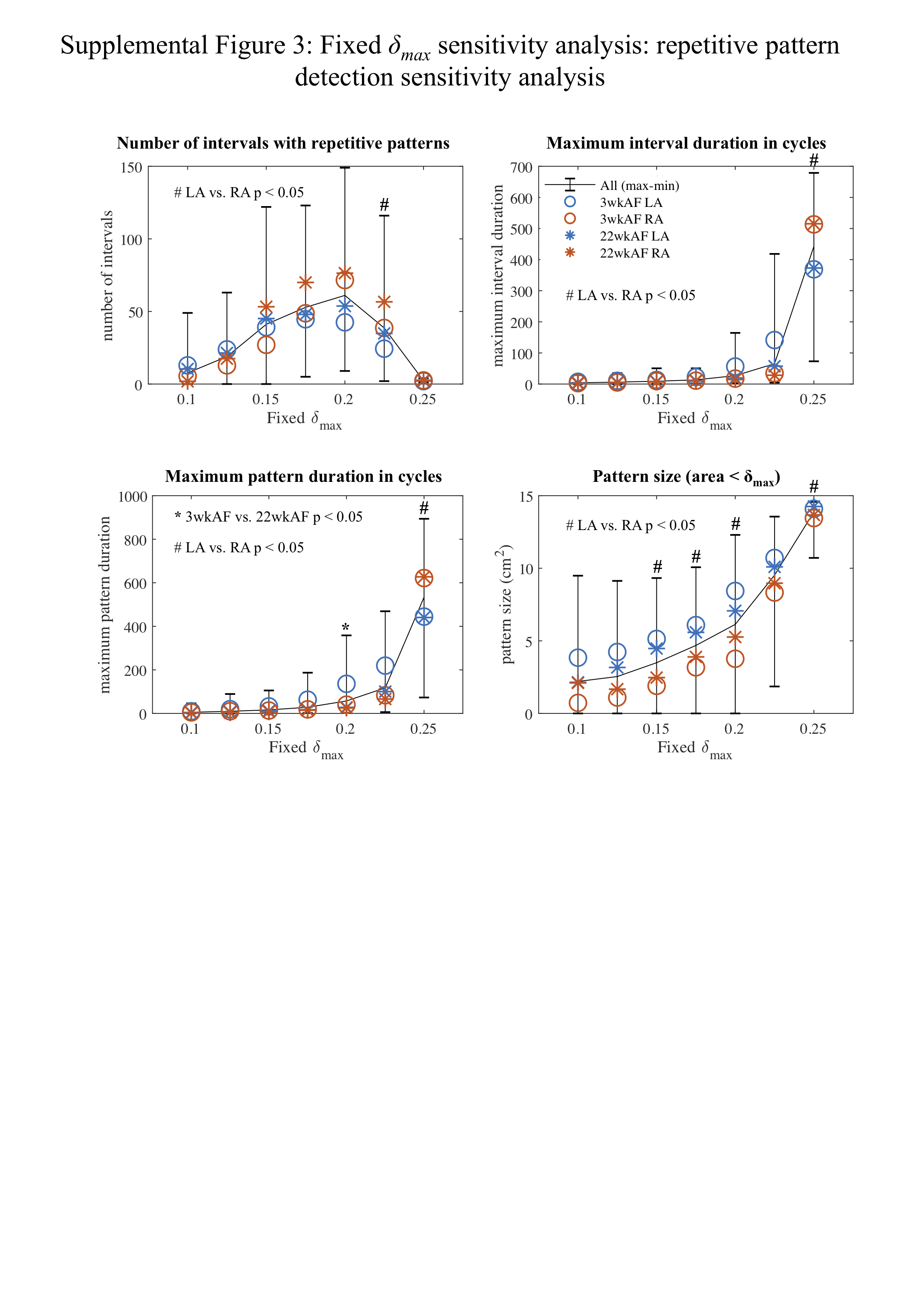

Supplement: Supplementary file 4 [file Image_3.JPEG]

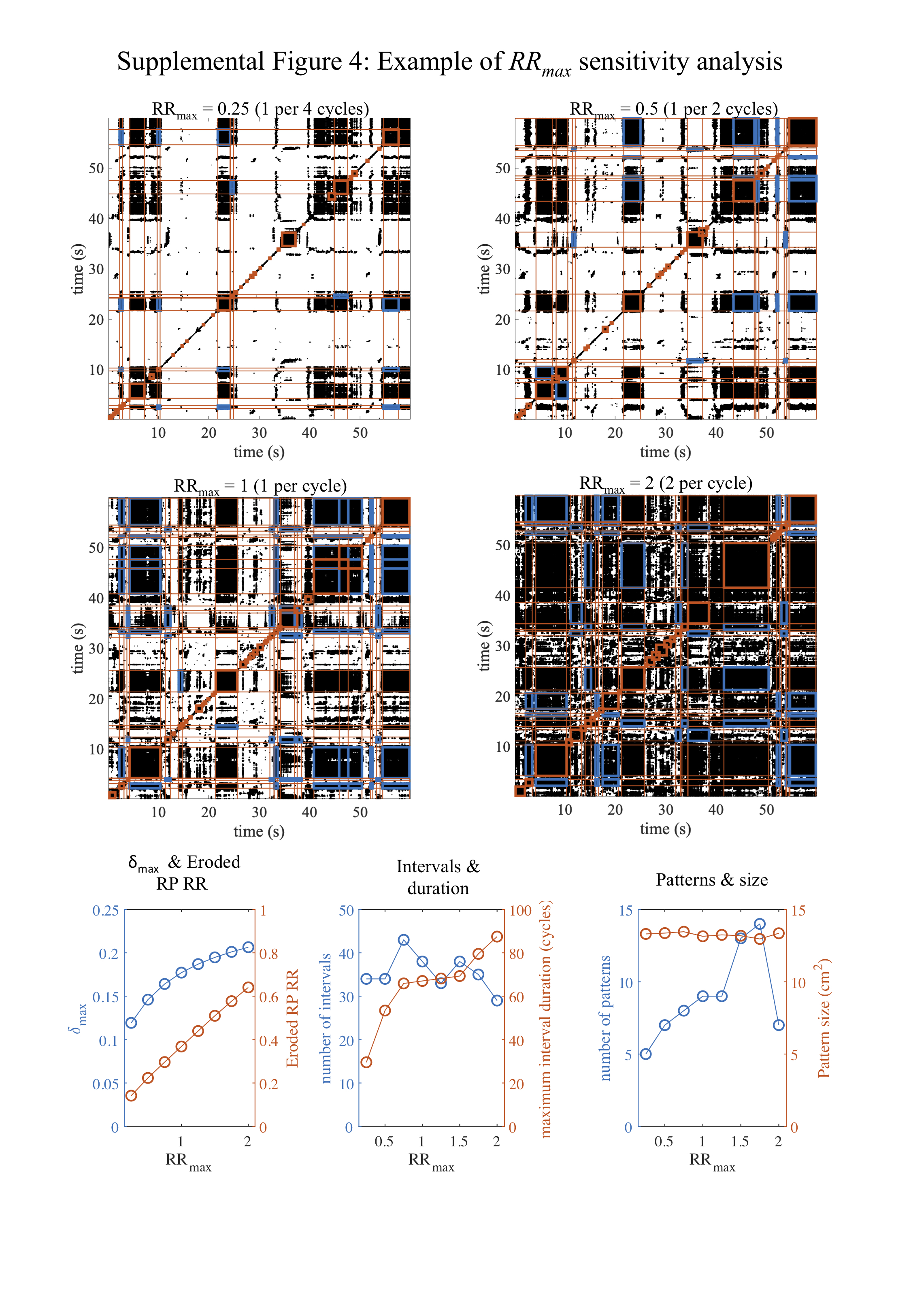

Supplement: Supplementary file 5 [file Image_4.JPEG]

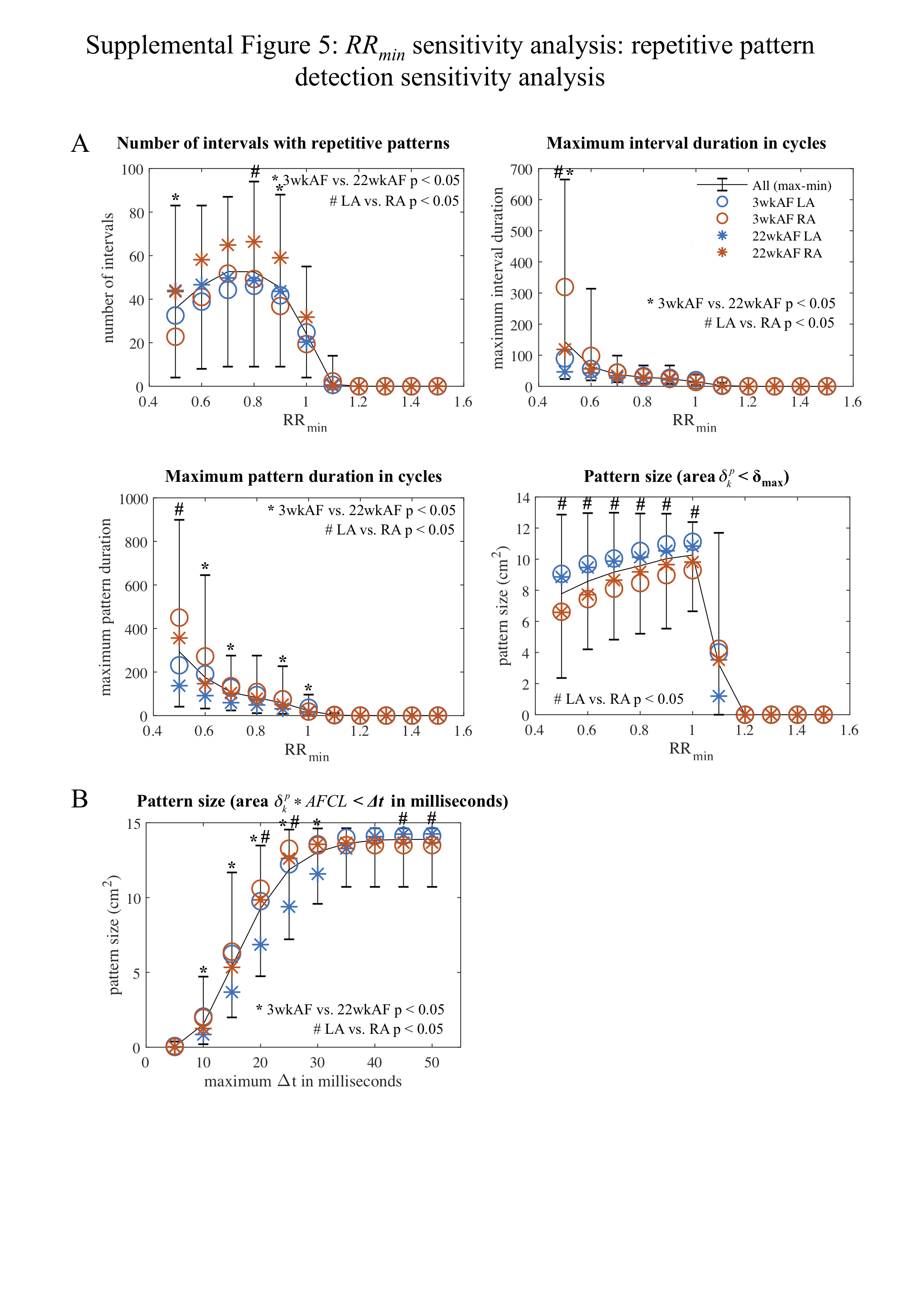

Supplement: Supplementary file 6 [file Image_5.JPEG]
